# Supplementary material for: Are carbon emissions trading and green financial instruments synergistic? -Comprehensive quantitative research based on content analysis
Source: PLoS One. 2024 Mar 7;19(3):e0298601. doi: 10.1371/journal.pone.0298601 (PMC10919601; doi:10.1371/journal.pone.0298601)
Supplement: S1 Data — (ZIP) [file pone.0298601.s001.zip › Data/地级市Z_F工作报告.zip!/░▓╗╒/┘±╓▌╩╨/└┤╫╘╣1⁄2╓┌║┼í╛┬φ┐╦╩2╛▌═°í┐_2015.docx]

各位代表：
　　现在，我代表市人民政府，向大会报告政府工作，请予审议，并请市政协委员和其他列席人员提出意见。
　　2014年工作回顾
　　过去的一年，面对复杂多变的宏观经济环境，在中共亳州市委的坚强领导下，在市人大、市政协的监督支持下，市政府紧紧依靠全市人民，认真贯彻落实党的十八大、十八届三中、四中全会和习近平总书记系列重要讲话精神，以稳中求进、好中求快为基调，以改革创新、扩大开放为动力，统筹做好稳增长、调结构、促改革、惠民生的各项工作，保持了经济社会持续健康发展的良好态势。
　　初步预计，全市地区生产总值850.5亿元，同比增长7.8%；全部财政收入115.7亿元、增长11.8%，其中地方财政收入72.7亿元、增长12.9%；社会消费品零售总额388.4亿元，增长13%；城镇、农村常住居民人均可支配收入分别增长10%和12%；节能减排完成省下达目标任务。
　　（一）各项改革力度进一步加大。深入推进行政体制改革，市级行政审批事项由366项减少到84项、减少77%，市级涉企收费清单编制完成，市县（区）工商、质检、食品药品行政管理体制和监管体制改革任务顺利完成，市公共资源交易"一委一局一中心"成功组建。全面落实注册资本金认缴制，推进工商注册便利化，全年新增企业5950家、增长51.6%。组建市建安投资控股集团，成功发行18亿元企业债。争取国开行为棚户区改造授信67亿元，占全省1/10。市县区政府及部门财政预决算和"三公"经费信息全部公开，政府购买服务工作有序开展。农信社改制全面完成，在全省率先实现村镇银行县区全覆盖，6家企业在省股权托管交易中心成功挂牌，亳州市中药材商品交易中心获批筹建。中小学教师职称省级改革试点工作稳步推进，县级公立医院综合改革和基层医改成果进一步巩固。我市顺利获批省工矿废弃地复垦利用试点单位，涡阳县完成农村土地确权登记颁证工作。
　　（二）经济增长基础进一步夯实。完成固定资产投资650.9亿元、增长20.2%，其中工业投资242.6亿元；海创新型节能建材等68个亿元以上项目基本建成，板集电厂等165个亿元以上项目加快建设，升级改造国省干线公路96公里，完成电网建设投资5.3亿元，济祁高速亳州段工程进展顺利，商杭高铁亳州段即将开工建设，亳州机场选址已经确定。全面推行精准招商，实际到位市外内资1000亿元、增长22%，新引进亿元以上项目102个、其中工业项目82个。5个省级开发区和亳芜现代产业园区加快建设。新增进出口实绩企业22家，生产型外贸企业实现进出口额3.7亿美元，实际利用外商直接投资6.1亿美元、增长27%。成功举办2014年国际（亳州）中医药博览会暨第30届全国（亳州）中药材交易会，签约金额199.6亿元。
　　（三）结构调整步伐进一步加快。预计，三次产业结构为24.1：40.1：35.8；与去年相比，一产占比下降0.5个百分点，三产占比上升0.9个百分点。开展"千名干部联系服务千家企业活动"，制定支持中小微企业的具体措施，大力促进实体经济发展。有效引导信贷投放，金融机构新增贷款118亿元，其中新增中小微企业贷款51.5亿元。全力支持重点产业发展，十大产业中的现代中药、食品制造和电子信息产业等产值均增长20%以上；新增规模工业企业175家，实现规模以上工业增加值224亿元、增长10.2%；古井贡酒股份有限公司经营业绩逆势上扬，收入增幅位居全国同行业前列。加快发展现代农业，粮食总产达93.4亿斤、实现"十一连丰"，亳州市和涡阳县、蒙城县被授予"全国粮食生产先进单位"称号；新增农民合作社717个、家庭农场892个，规模以上农产品加工企业产值增长15%；完成农田水利基本建设投入10.6亿元，改造中低产田13.8万亩，新增旱涝保收面积72万亩，蒙城县被列为全国深化小型水利工程管理体制改革示范县。稳步提升第三产业，实现旅游总收入88亿元，增长14.8%；完成房地产开发投资209.1亿元，增长40.2%；净增限额以上商贸流通企业70家，新建和改造农家店、直营便利连锁店109个。新认定高新技术企业7家、省级企业技术中心3个，新建院士工作站2个，新增授权专利857件，实现高新技术产业产值256.2亿元、增长20.9%。国家中药材产品质量监督检验中心开工建设，谯城区出口中药材质量安全示范区升级为国家级出口食品农产品质量安全示范区。
　　（四）城乡人居环境进一步改善。预计，城镇化率达36%，比上年提高1.6个百分点。强力推进城乡建设，在市中心城区实施建设项目247个，10项重点工程有序推进，市南部新区污水处理厂建成运行；全市新投入公交车280辆、出租车220辆；三县建成区面积达77平方公里，集聚人口75万人；加固改造农村公路危桥137座，改造县乡公路274公里；新增全国小城镇建设发展重点镇5个，美好乡村第一批26个省级重点示范村通过验收，第二批28个省级重点示范村建设有序推进；利辛县、蒙城县分获省皖北片区美好乡村评比第2名、第3名，被评为省美好乡村建设先进县。扎实推进城乡文明创建，深入开展"千名干部进网格"活动，开工建设市数字化城管系统，在市中心城区通过"随手拍"平台解决问题6万多个，出新小区楼道面积15.8万平方米，设置便民公告栏4676块；扎实开展"三线三边"环境治理，大力实施农村清洁工程和千万亩森林增长工程，清理垃圾22.4万吨、河道4700公里，完成人工造林13.5万亩，建设绿色长廊626公里；我市荣获第三届"安徽省文明城市"、第二届"安徽省未成年人思想道德建设工作先进城市"称号，蒙城县获"安徽省文明县"称号，涡阳县、利辛县获"安徽省文明县城"称号，谯城区获"安徽省文明城区"称号。推进大气污染防治、污染减排和资源节约集约利用，淘汰燃煤锅炉192台、黄标车和老旧车辆2.6万台，完成63家养殖场污染治理任务，创建国家级生态乡镇3个，我市成功列入国家创建新能源示范城市试点。
　　（五）和谐稳定大局进一步巩固。全市民生支出达183.9亿元、占财政支出的80.8%，32项民生工程完成年度任务。新开工建设保障性安居工程47246套，基本建成25815套；新增城镇就业5.2万人，城镇登记失业率控制在3.6%以内；城乡居民养老保险参保338.7万人，城市和农村低保标准、农村五保供养标准均提高10%以上；新建、改扩建农村敬老院20所，农村五保集中供养能力达70.4%；1175户渔民全部实现上岸定居，86万农村居民和3.5万师生饮水安全问题得到解决，老村干、老民办教师、老放映员、老公路养护工、老农技员、老兽医、老拖拉机手等"老字号"群体生活切实改善。全市教育经费实际投入39.8亿元，高考本科达线率达50.3%、高出全省8.6个百分点，亳州幼师新校区投入使用，亳州师专升本工作有序推进，亳州职业技术学院专升本班填补了我市无全日制本科教育的空白；蒙城县、涡阳县争创全国义务教育基本均衡发展县顺利通过国家级验收，蒙城县在皖北三市中首个获得"安徽省教育强县"称号；编写国学经典教材，扎实开展国学经典进校园活动。基本医保覆盖面不断巩固，全市新农合参合率98.7%，市全科医生培训中心、市医疗废物处理中心投入使用，30家乡镇卫生院、社区卫生服务中心设立示范中医科。市图书馆建成开放，开展各类文艺演出2911场，放映农村公益电影15495场，《老子传说》列入国家非物质文化遗产名录。全民健身运动广泛开展，华佗五禽戏进一步普及。人口自然增长率为8.5‰。妇女儿童、老龄、慈善、红十字、残疾人等事业持续发展，民族、宗教、外事、侨务、对台、物价、供销、统计、气象、防震、档案、地方志和无线电管理工作取得新成绩。全面落实"分管领导+主管单位+属地"信访工作责任机制，省以上交办信访案件按期办结率达100%。深入实施"六五"普法规划，有效处置各类突发事件，大力整治安全生产隐患，着力保障食品药品安全，依法打击各类违法犯罪，社会大局保持和谐稳定。全民国防教育、国防动员、人民防空和双拥优抚安置工作进一步加强，驻亳部队和民兵预备役人员在地方经济社会发展中作出了重要贡献。
　　（六）政府自身建设进一步加强。扎实开展党的群众路线教育实践活动，认真贯彻"三严三实"要求，着力整治"四风"积弊，切实解决群众反映强烈的突出问题，政府工作作风进一步转变。严格执行中央八项规定和省委、市委三十条规定，全市"三公"经费下降28.6%。坚决执行人大决定决议，自觉接受人大法律监督和工作监督、政协民主监督和群众社会监督，办复人大代表议案9件、建议204件和政协委员提案309件，满意率均达96%以上。继续开展发展环境优化年和民主评议百名科长活动，认真做好政务公开工作，切实加强"12345，电话找政府"热线平台建设，探索推进政府服务管理信息化，试点建设市网上办事大厅，市政府工作群众满意度调查成绩居全省第1位。认真落实党风廉政建设责任制，强化行政监察和审计监督，政府系统廉政建设进一步加强。
　　过去的一年，成绩来之不易。这是省委、省政府和市委正确领导的结果，是市人大、市政协大力支持的结果，是全市广大干部群众锐意进取、奋力拼搏的结果。在此，我代表市人民政府，向广大工人、农民、知识分子、干部、离退休老同志，向驻亳解放军指战员、武警官兵和政法干警，向各民主党派、各人民团体和各界人士，向中央和省驻亳单位，向所有关心支持亳州建设与发展的同志们、朋友们，表示崇高的敬意和衷心的感谢！
　　在肯定成绩的同时，我们也清醒地看到，我市经济社会发展中还存在不少困难和问题，主要是：地区生产总值、财政收入、进出口总额、农村常住居民人均可支配收入等指标增幅没能达到年度预期目标；土地节约集约利用水平不高，征迁政策执行不一，私搭乱建依然存在，项目落地难问题没有得到根本解决；有效金融供给和服务不足，企业特别是中小微企业融资难、融资贵；政府性债务进入还贷高峰期，偿债压力和财政收支平衡难度加大；社会事业发展滞后，基本公共服务均等化程度较低。同时，个别政府部门和工作人员还不同程度存在"四风"问题，发展环境有待进一步优化。对此，我们一定增强忧患意识、责任意识，强化问题导向、底线思维，以更大的决心、更有效的措施认真加以解决。
　　2015年目标任务
　　今年是全面深化改革的关键之年，是全面推进依法治国的开局之年，也是全面完成"十二五"规划的收官之年。当前，我市既面临"三期叠加"的普遍矛盾，也面临国家继续支持中部地区崛起、中原经济区建设和省加快推进皖北地区又好又快发展等重大机遇。只要我们把思想和行动统一到中央和省委、市委决策部署上来，自觉认识新常态、适应新常态、引领新常态，就一定能牢牢把握工作主动权；只要我们深刻领会新常态下做好政府工作的新要求，增强加快转变经济发展方式的自觉性和主动性，就一定能实现稳中提质、稳中求好；只要我们紧密结合亳州实际，发挥比较优势，积极主动作为，就一定能开创全市经济社会发展新局面。
　　今年政府工作的总体要求是：全面贯彻落实党的十八大、十八届三中、四中全会和习近平总书记系列重要讲话精神，坚持以经济建设为中心，坚持加快科学发展不动摇，坚持稳中求进工作总基调，主动适应经济发展新常态，狠抓改革攻坚，扩大对外开放，推进结构调整，保障改善民生，维护社会稳定，在活力亳州、美丽亳州、幸福亳州现代化建设征途上迈出新步伐。
　　经济社会发展的主要预期目标是：地区生产总值增长8%以上，财政收入增长10%左右，固定资产投资增长15%左右，社会消费品零售总额增长11%左右，进出口总额增长10%，城镇、农村常住居民人均可支配收入分别增长9%和10%，城镇登记失业率控制在4.2%以内，人口自然增长率控制在9‰以内，节能减排完成省政府下达的年度目标任务。
　　围绕上述目标，重点做好以下八个方面的工作：
　　（一）着力推动重点领域改革取得新突破。
　　坚持问题导向，以政府自身革命推进重点领域和关键环节改革，最大限度地释放改革红利、激发市场活力。
　　大力推进行政管理体制改革。扎实推进行政审批制度改革，加快建立市、县区政府权力清单、责任清单和涉企收费清单，尽快全面公布运行，并根据运行情况，进一步精简和取消一批行政审批事项。全面清理非行政许可审批，大幅减少前置审批。继续推进工商登记制度改革，探索试行企业登记注册工商营业执照、组织机构代码证和税务登记证"三证合一"。深化为民服务全程代理，完善网上办事大厅功能，逐步实现市、县（区）、乡（镇）三级实体性行政办事大厅向网上办事大厅迁移。加大政府向社会购买公共服务力度。推动公务用车制度改革。
　　继续深化农村综合改革。全面完成省政府下达的农村土地承包经营权确权登记颁证试点工作，大力推广"土地托管"、"一块田"模式，力争流转土地面积达340万亩。深化农村金融改革，鼓励符合条件的县区农商行互设分支机构，拓宽涉农信贷渠道。继续深化小型水利工程管理体制改革。加快农业社会化服务体系建设，积极发展多种形式适度规模经营。
　　着力抓好财税金融等领域改革。全面落实中央和省财税体制改革要求，有序推进"营改增"，认真清理规范税收优惠政策，实行全面规范、公开透明的预算管理制度。所有使用财政资金的部门，除涉密信息外都要公开预决算和"三公"经费信息。严格政府债务管理，防范债务风险。加快地方金融体系建设，支持村镇银行向乡镇延伸网点，积极引进域外银行、保险、证券机构入驻亳州。推进广印堂等企业上市进程，鼓励引导更多企业在"新三板"和省股权交易中心挂牌，做好亳州市中药材商品交易中心筹建工作。全面推进医药卫生体制改革，启动城市公立医院改革，巩固基层医改和县级公立医院改革成果，所有公立医院实行基本药品零差率销售。
　　（二）大力促进民营经济快发展。
　　始终把民营经济作为重要抓手，着力破解资金、土地等要素制约，不断提高经济发展的内生动力。
　　破解资金难题。加强银政担险企合作，努力扩大信贷投放，力争全年新增贷款130亿元以上。鼓励商业银行加大对民营经济的信贷支持力度，确保中小微企业贷款增量不低于上年。发挥财政资金引导作用，通过贷款贴息补助、提供融资担保和贷款风险补偿等多种形式，着力缓解融资难、融资贵的问题。
　　节约集约用地。坚持严控总量、盘活存量，全面清理闲置低效用地，大力推进城乡建设用地增减挂钩工作。出台相关政策，引导社会资本投入标准化厂房建设，促进土地节约集约利用。对利用历史遗留乡镇工业用地生产经营的民营企业，在符合规划的前提下，每年安排一定的专项指标，帮助其实现权证化。
　　促进公平发展。深入开展"千名干部联系服务千家企业"活动，全面落实支持民营经济发展的政策措施。强化为企服务网上平台建设，清理不必要的证照和资质、资格审批，着力打破"玻璃门"、"弹簧门"、"旋转门"等障碍。按照"非禁即入"原则，对民营企业在各方面与国有、集体企业一视同仁，支持民营企业以独资、合资、参股、特许经营等方式参与经营性基础设施、公益事业项目建设和经营管理。降低准入门槛，引导个体工商户、家庭农场转型为个人独资企业、合伙企业或有限责任公司等。大力发展会展经济，积极搭建产供销平台，加强品牌培育，支持企业开拓市场。实施"小巨人"培育提升计划，重点培育100家工业企业、50家商贸企业和50家农业产业化龙头企业，力争全年新增个体工商户1.8万户、私营企业3000家。
　　（三）坚定不移实施开放带动战略。
　　适应新常态，把握新要求，坚持对内对外开放并举，以开放促进经济结构调整，以调整促进产业优化升级。
　　把招商引资作为对外开放的首要任务。积极应对国家清理规范税收等优惠政策带来的新挑战，通过设立产业发展基金、提供标准化厂房、优化投资环境等，不断改进招商引资方式。紧紧围绕十大重点产业发展目标，坚持招大引强引新，坚持一、二、三产业一起上，国有、集体、民营一起引，大力实施驻点招商、小分队招商、精准招商、借会招商，加强与外企、央企、省企、知名民企合作，对在手招商线索紧盯不放直至推动落地，力争全年引进内资增长20%、外商直接投资增长10%。坚持以质量和效益为中心，统筹引资、引技与引智，坚决摒弃拼土地、拼资源、拼环境，坚决防止引进落后产能和污染企业。
　　坚持工业强市战略不动摇，加大工业招商力度，引导企业采取资产重组、战略合作、产业链招商等方式，引进一批关联性强的工业项目，力争全年新增规模以上工业企业100家，产值超亿元企业达到180家。突出抓好现代中药产业招商，努力在成药制造、中医医疗保健和医疗器械生产上实现突破，确保实现医药制造业产值200亿元。推进古井配套产业园等重点项目建设，力争古井集团实现销售收入60亿元，全市食品制造及农产品加工业产值达到300亿元。依托江淮安驰、中联重机、怡科电子等骨干企业，着力推动汽车农机及零部件、电子信息、劳动密集型装备制造业规模化发展。大力推进工业化、信息化深度融合，培育发展新材料、3D打印等新兴产业，力争战略性新兴产业实现产值260亿元、高新技术产业产值增长20%以上，新增高新技术企业6家、省级创新型试点企业4家，新培育民营科技企业10家。完成发明专利申请800件，新增省著名商标10件。
　　加快现代服务业和农业等领域引资步伐。发挥亳州旅游资源优势，吸引旅游产业战略投资者来亳创业，着力打造"曹操运兵道-花戏楼-大寺闸"精品线路，全力推进曹操运兵道、蒙城博物馆、利辛印象江南等4A级旅游景区创建工作，引导发展乡村旅游，全年创建3A级以上景区3家、3星级以上农家乐5家、3星级以上旅游饭店2家，力争旅游总收入达100亿元。积极推行多种形式的土地承包经营权流转，吸引市外资金发展农业规模化经营和设施农业。加大企业总部招商力度，吸引国内外有实力的大企业在我市设立地区总部和研发中心、采购中心、会展服务中心等。
　　加快推进园区转型升级。继续推进道路、电力、通讯、排污等基础设施和生活类服务设施建设，完善园区配套功能，提升项目承载能力。加快现代中药、劳动密集型装备制造、电子信息、农产品加工等特色园区建设，形成一批主业突出、特色鲜明、核心竞争力强的园中园。把项目落地作为开发区建设的重中之重，运用政策、法律等多种手段，强力推进征地拆迁，集中力量保开工、快建设、早投产。严格执行投资强度、亩均税收、建设容积率等政策规定，着力提高投入产出效益。
　　大力发展外向型经济。加快培育外贸主体队伍，积极支持中药材、农副产品、汽车微卡等优势产品出口，鼓励企业扩大先进技术设备、原材料等产品进口。鼓励有条件的企业设立境外营销机构，积极开拓国际市场。重点选择与我市经济互补性强、产业关联度高的国家和地区，建立友好城市；深化与芜湖等市的结对合作，提高合作成效。加强对外开放窗口建设，推进贸易便利化，全面提升外向型经济水平。加大城市品牌推广力度，办好2015年国际(亳州)中医药博览会暨第31届全国(亳州)中药材交易会。
　　（四）不断提高内需拉动力。
　　保持经济稳定增长是今年政府工作的主要任务，我们必须坚持投资、消费两手抓，更好发挥投资的关键作用和消费的基础作用。
　　多管齐下扩大有效投资。围绕国家产业政策和投资导向，坚持以资源换项目、以市场换项目，重点谋划一批打基础、管长远的基础设施和产业项目，谋划一批惠民生、促和谐的生态环保和社会事业项目，全年谋划储备项目投资额3000亿元以上。加强项目申报、推介和招商工作，形成滚动增加、梯次实施的良性循环。加快建设一批重点项目，开工建设商杭高铁亳州段、济祁高速利辛至淮南段、利辛夏湖220千伏输变电工程等基础设施项目，加快亳州机场、涡河航道整治、引江济淮亳州段等项目前期工作，确保济祁高速永城至利辛段、省道307线西阳至蒙城段、绕城快速通道西线主体工程顺利推进。重视发挥政府投资的引导作用，吸引社会资本投入战略性新兴产业和城建、教育、体育、医疗卫生、健康养老等领域。
　　多方配合加快项目建设。坚持经济工作项目化、项目工作责任化，完善落实政府投资城建项目法人责任制、合同管理制、工程变更管理制、竣工验收管理制和公共资源交易监督管理制、审计监督制等6项制度，进一步明确相关部门和地方在项目前期工作、征地拆迁、招标投标、组织实施、监督管理等方面的责任。坚持"定责任主体、定工作目标、定完成时限"，加强部门联动配合，坚决破除项目实施中的各种障碍。坚持以项目看实绩、以项目看水平、以项目看担当，修订完善项目建设考核办法，严格兑现奖惩，积极推动126个未投产项目、52个未达产项目尽快投产达效。
　　多措并举促进大众消费。着力扩大城乡居民收入，落实分配制度改革政策，稳步提高最低工资标准，健全职工工资正常增长机制，积极推动落实带薪休假制度，夯实居民消费基础。抓住国家放开服务业准入限制和"营改增"扩围的有利时机，积极发展健康养老、旅游休闲、体育健身等生活性服务业，加快发展信息服务等生产性服务业，不断改善消费环境。加强市场调控，保持价格总体水平基本稳定。深入实施城市商业网点规划，加快商贸市场建设。着力推进亳州万达广场、大唐国际城等重点项目建设，积极推动华润苏果等企业在市区和三县扩大布点，力争新增限上商贸流通企业50家。加强便民服务体系建设，年内建成社区菜市场、便利店、早餐店、家政服务点等商业网点100家。扶持发展电子商务，引导电子商务产业园扩容升级，支持中药材电子商务平台建设，充分发挥农业物联网示范点带动作用，力争阿里巴巴亳州馆销售额突破3亿元。积极培育新的消费热点，推动旅游、绿色、大众餐饮等新型消费。
　　（五）加快推进农业现代化。
　　坚定不移走中国特色新型农业现代化道路，加快转变农业发展方式，不断提高土地产出率、资源利用率、劳动生产率，实现集约发展、可持续发展。
　　提高农业综合生产能力。坚定不移地抓好粮食生产，大力实施粮食高产创建活动，粮食总产稳定在90亿斤以上，努力实现"吨粮市"创建目标。加快推进中储粮亳州直属库新建20万吨粮食仓储及配套设施项目，抓好市级5万吨储粮粮库项目建设。新增设施蔬菜8万亩，力争蔬菜面积稳定在190万亩以上。大力发展肉牛、肉羊等规模养殖，新增各类规模养殖场（户）1000家。强化农产品质量安全监管，新增无公害农产品产地、绿色食品原料基地15个，新增农业"三品一标"认证产品30个。大力提升农业机械化装备水平，力争全市农机装备总动力达850万千瓦。建成市级农产品质量安全检测中心。加大对农业产业化龙头企业的扶持力度，引导城市工商资本重组、改造龙头企业，龙头企业引领家庭农场，家庭农场带动千家万户。加快发展农产品深加工业，力争产值亿元以上企业达到100家。扎实推进农产品加工园区建设，每个县区确保规划建设1个农产品（食品）加工园区。深入推进农村新型经营主体规范发展，力争年内家庭农场发展到1500家、农民合作社发展到5400家。
　　加强农村基础设施建设。全面实施"八小"水利工程和小农水重点县项目，改造泵站1415千瓦，新建加固小型水闸103座，整治河沟587条，新建、修复机电井3115眼。实施阜蒙河、武家河、北淝河等中小河流治理工程，做好西淝河、北淝河洼地治理工作。加快气象防灾减灾标准化乡镇和示范村建设。巩固农村饮水安全工程建设成果，新解决96.6万农村居民和2.6万学校师生安全饮水问题。改建农村公路200公里。
　　开展农村土地综合整治。认真抓好2015年城乡建设用地增减挂钩拆旧项目区储备库建设，确保全年完成组卷2万亩，实施1万亩拆旧项目区工作；编制完成工矿废弃地复垦利用规划，抓好工矿废弃地复垦利用试点实施工作，确保复垦利用土地不少于3000亩；全面完成高标准基本农田建设任务；强力推进省级投资土地整治项目、土地整治示范项目村庄拆迁复垦工作；力争全年补充耕地1.2万亩。
　　大力推进美好乡村建设。坚持群众的事自己议、自己定、自己干、自己管，紧密结合群众实际需求，科学确定村庄布局和规模，全面完成第二批28个省级重点示范村建设任务，加快推进第三批31个省级重点示范村建设。把保障农民基本生产生活条件放在首要位置，计划投资6.6亿元，建设项目460个，重点抓好垃圾污水处理、村庄道路、危房改造、绿化亮化等配套设施建设。积极鼓励和支持农民返乡创业，引导发展特色产业，按照"一村一品"要求，着力培育59个特色产业村。
　　（六）积极推进新型城镇化。
　　城镇化是现代化的必由之路，我们要按照国家和省统一部署，努力走出一条符合亳州实际的新型城镇化道路。
　　建设中心城区和三县县城。优化中心城区空间布局，完善城市整体功能，加快推进供电、教育、卫生、环卫、绿地等专项规划编制工作。加快棚户区、城中村改造步伐，建设保障性安居工程26388套。推进南部新区综合交通枢纽站前期工作，建设中心城区周边汽车客运站，适当增加市区公交车，千方百计增加中心城区停车泊位。坚持安置优先，依法做好土地房屋征迁工作，确保城市建设顺利进行。深入开展县城"三治、三增、三提升"行动，全面加强道路管网、电力通讯等基础设施建设，吸引社会资本采取PPP模式等多种形式参与城市供水供气、公共交通、污水垃圾处理等设施建设和运营，着力完善学校、医院等公共服务设施，努力提升县城功能。支持蒙城县做好撤县设市相关工作。
　　增强城镇承载能力。扎实推进户籍制度改革，全面推行居住证制度，引导农业人口有序向城镇转移并享有基本公共服务，努力解决进城农民留得下、住得稳、生活得好的问题。统筹发展资本密集型、技术密集型和劳动密集型产业，大力发展就业容量大的服务业，促使城镇与产业发展良性互动。坚持以就业促创业，实施更加积极的就业政策，健全公共就业服务体系，重点做好高校毕业生、农村转移劳动力、下岗失业人员、退役军人就业工作，确保新增城镇就业岗位4.5万个，转移农村富余劳动力10万人。引导各县区因地制宜发展特色产业，不断壮大县域经济。
　　加强城镇规划和管理。坚持"规划一张图、城乡全覆盖"，探索"多规合一"模式，做到一个市（县、区）一张图、一张蓝图干到底。根据区位优势、资源禀赋、产业特色，完成中心集镇总体规划修编，完善乡镇规划建设管理办法，落实规划管理责任。全市所有未实施的行政村都要完成中心村布点规划，启动一期占地至少10亩的新村建设，并根据需要滚动发展，加强农村住房建设管理，引导农民规范建房，着力清除违法用地。把依法拆违控违作为中心城区和三县县城加强城市管理的首要任务，坚决制止私搭乱建行为，严肃追究相关责任人责任。
　　（七）切实加强生态环境建设。
　　生态文明关系人民福祉，我们必须以更大的力度抓好污染防治、环境保护和生态建设，让广大人民群众在更好的环境中工作和生活。
　　解决城乡环境问题。紧紧围绕"九城同创"工作目标，着力解决好群众反映强烈的城乡环境问题，切实回应群众关切。深入开展"千名干部进网格"工作，强化文明创建"随手拍"平台推广运用，进一步完善问题发现交办、销号管理和检查考核机制。深入开展农村清洁工程和"三线三边"治理，加大增绿、增彩、增果工作力度，着力改善城乡环境。
　　提升中心城区品位。实施"地净"工程，以城区道路清洁为重点，加大清扫监管力度，推进道路清洁治理全域覆盖。实施"路畅"工程，积极开展"文明交通，人人有责"主题活动，实施以互联网为技术基础的"智慧交通"工程，强化道路交通科学管理，启动城区物流企业综合整治，完成涡河人民大桥维修加固工程，加快推进涡河建安大桥建设前期工作，打通利辛路、人民路西段、汤王大道北段等"断头路"。实施"水活"工程，加快贯通涡河城区段、宋汤河、凤尾沟、亳城河水系，让涡河水在城区活起来。实施"天蓝"工程，加大雾霾监测预警和治理力度，强化工业废气、汽车尾气、施工扬尘、城市道路扬尘等专项整治，抓好燃煤锅炉、油气回收、垃圾焚烧、秸秆焚烧、餐厨油烟污染等治理工作，淘汰"黄标车"及老旧车辆。实施"林拥城"工程，围绕主城区200平方公里规划边界和京九铁路城区控制区，启动建设高标准林带，着力构筑城市绿色城墙；加强城市主干道和街头游园绿地建设，做到见缝插绿、按需补绿、拆墙透绿、应绿尽绿，着力打造"城在林中、林在城中"的生态景观；深入实施千万亩森林增长工程，完成人工造林14万亩，建设绿色长廊480公里。开工建设市植物园。加大噪声污染治理力度，为城乡居民营造宁静的生活环境。
　　抓好节能和生态保护工作。完善节能预警调控机制，切实抓好工业、建筑、交通、公共机构和居民生活等领域的节能工作。积极探索现代农业与太阳能等新能源产业结合，大力发展分布式光伏发电，推进生物质发电项目建设，加快创建国家新能源示范城市。落实最严格的环境保护措施，推行生态环境损害责任终身追究和赔偿制度，大力促进绿色低碳发展。建立吸引社会资本投入生态环境保护的市场化机制，鼓励各类投资主体以多种形式参与生态环境建设。
　　（八）加强以保障和改善民生为重点的社会建设。
　　坚持守住底线、突出重点、完善制度、引导舆论的基本思路，多些雪中送炭，更加注重保障基本民生，更加关注困难群体生活，更加重视社会大局稳定，让发展成果更多更公平地惠及全市人民。
　　切实加强社会保障。继续做好省安排的民生工程各项工作。加快完善城乡社会保障体系，扎实推进老农保与城乡居民保险制度衔接工作，推动企业全员足额参保，全面实施城镇医疗保险省内双向异地联网结算，实现城镇居民大病保险全覆盖，加快推进社会保障"一卡通"建设。持续加强和改进社会救助工作，进一步清理和规范城乡低保，保障困难群体基本生活，城市、农村低保和五保供养标准分别提高10%以上；认真落实困难群众临时生活救助制度，为困难群众兜底线、救急难。推进社会养老服务体系建设，新建、改扩建敬老院14所，新增床位1000张以上。
　　大力发展教育卫生事业。优化城乡教育资源布局，制定并实施中心城区学前教育及义务教育布点专项规划。实施学前教育三年提升计划，努力解决城镇幼儿"入园难"问题。实施薄弱学校改造工程，促进义务教育均衡发展。实施千所村级小学提升工程，完成200所学校提升任务。落实困难学生就学扶助政策，确保不让一名学生因家庭经济困难而失学。在全市中小学深入开展国学经典教育。加强师德师风教育，探索建立城乡教师常态化互动交流机制。坚持产教融合、校企合作，大力发展职业教育。全力支持亳州师专升本工作，大力支持亳州职业技术学院创建省示范骨干高职技术院校。加快推进新农合医保体系建设，深入实施中医药能力提升工程，大力发展社会办医，加快推进市人民医院新院二期工程、华佗中医院新院建设。切实抓好人口计生工作，稳妥实施"单独二孩"政策，积极发展妇幼卫生事业，认真做好传染病防治工作。
　　统筹发展各项社会事业。加强公共文化设施建设，做好公共文化场馆免费开放工作，推动公共文化服务体系向基层延伸。繁荣地方文艺创作，传承发展非物质文化遗产，广泛开展送戏下乡、送戏到敬老院和城市文化广场活动。完善全民健身公共服务体系，促进群众体育向社区和乡镇延伸，推广普及五禽戏。加大农村扶贫开发力度，实施精准扶贫，着力解决农村贫困人口脱贫问题。做好民族、宗教工作，关爱留守儿童、留守妇女和留守老人，支持残疾人事业发展。加强统计、外事、侨务、对台、防震减灾、无线电管理和机关事务管理工作，推动广播电视、档案、地方志、红十字事业健康发展。持续深化全民国防教育，加强国防动员机构和能力建设，提升双拥共建水平，推进军民融合深度发展。
　　积极推进社会治理创新。深入开展评选道德模范和"身边好人"活动，大力培育文明新风。启动第二次全国地名普查，认真开展第五届社区居委会换届选举工作。积极推动社区管理体制改革试点。抓好法治宣传教育，推进公共法律服务体系建设，全面做好"六五"普法验收工作，着力健全社会信用体系。不断完善应急管理机制，加强消防安全管理，提升突发事件处置能力。坚持"党政同责、一岗双责"，加强安全生产监管，坚决遏制较大事故，杜绝重特大事故发生。强化食品药品安全监管，健全基层监管组织，确保人民群众饮食用药安全。认真落实"三位一体"工作责任机制，实施信访积案攻坚，依法及时就地化解矛盾。深入推进"平安亳州"建设，加大公安科技信息化建设力度，完善社会治安防控体系建设，依法预防和打击违法犯罪活动，确保社会和谐稳定。
　　加快建设法治政府
　　面对新形势、新任务和新要求，我们必须坚持党的领导，更多地运用法治思维和法治方式，加快建设职能科学、权责法定、执法严明、公开公正、廉洁高效、守法诚信的法治政府。
　　（一）全面推进科学行政。牢固树立科学发展观，强化科学意识，提高科学素养。坚持发展必须是遵循经济规律的科学发展，必须是遵循自然规律的可持续发展，必须是遵循社会规律的包容性发展，一切从亳州实际出发，不想当然作决策、拍脑袋定方案、凭经验办事情，不做违反科学的事、违反客观规律的事。全面正确履行政府职能，强化公共服务、市场监管、社会治理、环境保护等职责。树立正确的考核导向，完善符合科学发展要求的实绩考核评价体系。
　　（二）全面推进民主行政。坚持民主集中制，对涉及经济社会发展、与群众利益密切相关的重大事项，实行集体研究决定，不随意化安排、主观式屏蔽、选择性失聪。建立健全定期向人大报告工作、与政协沟通协商制度，主动征求人大、政协意见，广泛听取各民主党派、工商联、无党派人士和各人民团体意见，自觉接受新闻舆论监督和群众社会监督。大力推进政务公开，全面推进行政职权、财政预决算、公共资源配置、公共服务等重点领域信息公开，保障干部群众的知情权、参与权、选择权和监督权。加强"12345，电话找政府"热线平台建设，从细微处、身边事入手，切实解决人民群众最关心、最直接、最现实的利益问题。
　　（三）全面推进依法行政。把依法行政贯穿政府工作全过程，坚持依法行使权力，维护宪法法律权威，努力建设法治政府。自觉接受人大法律监督、政协民主监督，认真办理人大议案、代表建议和政协建议案、提案。建立健全依法决策机制，着力规范行政行为，严格按照法定权限和程序行使权力、履行职责。加大预算管理制度改革力度，切实做到预算之外无支出、预算报经人大审查批准。深化行政执法体制改革，提高执法和服务水平。建立政府法律顾问制度，健全行政复议案件审理机制，纠正违法或不当行政行为。推进政府权力流程规范化，实现行政权力与责任紧密挂钩、行政权力与主体利益彻底脱钩。坚持用制度管权管事管人，认真执行领导干部报告个人事项等制度，完善公共资源交易管理制度。加强行政监察，坚决治理和纠正损害群众利益的不正之风。加大审计工作力度，推进对公共资金、国有资产、国有资源和领导干部履行经济责任情况的审计监督全覆盖。切实加强党风廉政建设，严格落实中央八项规定和省委、市委三十条规定，自觉履行"一岗双责"，遵守廉洁自律各项制度，集中整治政府系统"四风"问题，以干部清正、政府清廉、政治清明赢得广大人民群众的信任、支持和拥护。
　　新常态的征程已经开启，新发展的号角催人奋进。让我们在中共亳州市委的坚强领导下，团结一致、苦干实干，奋力开创活力亳州、美丽亳州、幸福亳州现代化建设新局面！
　　《政府工作报告》名词术语解释
　　1．一委一局一中心--即公共资源交易监督管理委员会、公共资源招投标监督管理局和公共资源招投标交易中心。
　　2．家庭农场--以家庭成员为主要劳动力，从事农业规模化、集约化、商品化生产经营，并以农业收入为家庭主要收入来源的新型农业经营主体。
　　3．三线三边--"三线"即铁路沿线、公路沿线、江河沿线；"三边"即城市周边、省际周边、景区周边。
　　4．土地托管--土地流转的一种形式，是指部分不愿耕种或无能力耕种者把土地托给种植大户或合作组织，并由其代为耕种管理的做法。
　　5．新三板--即全国中小企业股份转让系统，是经国务院批准，依据证券法设立的继上海证券交易所、深圳证券交易所之后第三家全国性证券交易场所，主要为全国创新型、创业型、成长型非上市中小微企业发展服务。
　　6．三品一标--无公害农产品、绿色食品、有机农产品和农产品地理标志的简称。
　　7．"八小"水利工程--即"小水库、小泵站、小水闸、中小灌区、塘坝、河沟、机电井、末级渠系"等8类小型水利工程。
　　8．三治三增三提升--"三治"即治脏、治违、治乱；"三增"即增强功能、增加绿量、增进文明；"三提升"即提升规划水平、提升建设水平、提升管理水平。
　　9．PPP模式--即public-private-partnership的字母缩写，通常译为"公共私营合作制"，是指政府与营利性企业、非营利性企业，为了合作建设城市基础设施项目，或是为了提供某种公共物品和服务，以特许权协议为基础，彼此之间形成一种伙伴式的合作关系，并通过签署合同来明确双方的权利和义务，以确保合作的顺利完成，最终使合作各方达到比预期单独行动更为有利的结果。
　　10．九城同创--市委、市政府决定，从2014年起，在我市开展争创全国文明城市、全国未成年人思想道德建设工作先进城市、国家卫生城市、国家环境保护模范城市、全国社会管理综合治理优秀市、全国双拥模范城、国家园林城市、国家智慧城市和国家新能源示范城市等工作，简称"九城同创"。
